# Does Ultrasound Energy Applied During Phacoemulsification Influence the Thickness of Intraretinal Layers?

**DOI:** 10.3390/jcm14093049

**Published:** 2025-04-28

**Authors:** Slaven Balog, Marija Olujić, Antonio Kokot, Štefanija Kolačko, Damir Bosnar, Jurica Predović

**Affiliations:** 1 Ophthalmology Polyclinic Dr. Balog, Ivana Gundulića 36 b, 31000 Osijek, Croatia; slavenbalog@gmail.com (S.B.); molujic9@gmail.com (M.O.); 2 Faculty of Medicine Osijek, Josip Juraj Strossmayer University of Osijek, Josipa Huttlera 4, 31000 Osijek, Croatia; damir.bosnar@gmail.com (D.B.); jpredovi@gmail.com (J.P.); 3 Department of Ophthalmology, University Hospital “Sveti Duh”, Sveti Duh 64, 10000 Zagreb, Croatia; kolackostefy@gmail.com; 4 Department of Healthcare, University of Applied Health Sciences, Mlinarska Cesta 38, 10000 Zagreb, Croatia; 5 Faculty of Dental Medicine and Health Osijek, Josip Juraj Strossmayer University of Osijek, Crkvena 21, 31000 Osijek, Croatia

**Keywords:** cataract, macula lutea, optical coherence tomography (OCT), retina, phacoemulsification

## Abstract

**Background:** Phacoemulsification (PE) is a gold standard method of cataract surgery. PE causes structural changes in the macula of the eye. The aim was to determine the amount of ultrasound (US) energy applied during PE, the change in thickness of the intraretinal layers, and the correlation between the applied US energy and the change in the thickness of the intraretinal layers in five predefined areas of the macula. **Methods:** This prospective study included 102 eyes of 79 consecutive subjects without other eye or systemic diseases that can affect the eyes submitted to PE. The macular layer thickness was measured on the day of PE (day 0) and at 7, 30, and 90 days after PE using an OCT device. Recorded images were based on posterior pole asymmetry analysis. **Results:** Statistically significant correlations of applied US energy and the change in thickness of retinal layers were found 90 days after PE in 6 out of 10 macular layers in different macula regions. **Conclusions:** The results suggest interconnections between US energy spent during PE and the change in the thickness of individual retinal layers of the macula, in different ranges, unevenly, in five defined areas of the macula.

## 1. Introduction

Cataracts are still considered the leading cause of visual acuity reduction worldwide [1], often causing visual impairment in the elderly and infirm populations. Surgery in the early stages of the disease is usually an effective way to restore effective visual acuity and thus improve the quality of vision and life.

Ultrasound (US) assisted cataract surgery, i.e., phacoemulsification (PE), is nowadays the most common method of cataract surgery [2]. It has been shown that PE causes structural changes in the intraretinal layers, specifically in the area of the macula lutea [3,4], which is responsible for central visual acuity and part of peripheral vision. Any pathological event in the posterior pole of the eye might result in a loss of visual function and cause serious difficulties in the individual’s daily functioning. PE induces relative thickening of the macular intraretinal layers [3,4]. Macular thickness and the retinal nerve fiber layer have shown a significant increase after cataract surgery, with a maximum increase in retinal thickness one month after surgery, and a regression of this thickening six months after surgery [5]. Although, so far, the majority of articles have been focused on the foveola, the changes mentioned above occur and persist at the periphery of the macula [3,6,7]. The location of the changes within the macula could depend on the position of the surgeon’s hand and the position of holding the PE probe during the surgery (i.e., whether the surgeon is right-handed or left-handed) and could consequently result in statistically significant changes in the relative thickness of certain areas of the macula [3,8].

PE is considered the most frequently performed intraocular surgery in the world. The technique is based on US energy, which has two components. The first component is the mechanical cutting energy, which many believe is the only energy in PE. The second component is considered the sound wave of the US, which produces cycles. Those cycles interfere and result in the formation of small bubbles or cavities. It is most commonly performed on older patients with cataracts and is generally associated with good visual improvement [6,9,10,11,12]. In general, PE does not lead to clinically significant retinal changes.

**Figure 1:**
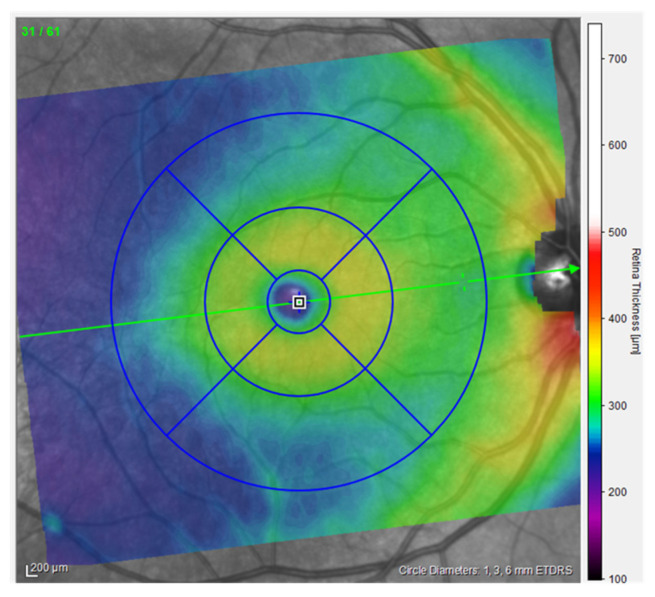
ETDRS view of the right eye, measurements from the participants (published with permission from Heidelberg Engineering GmbH).

**Figure 2:**
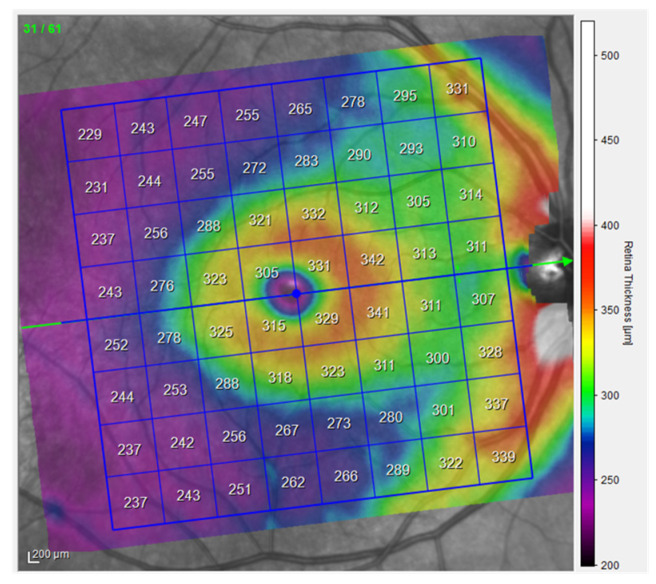
PPAA view of the right eye, measurements from the participants (published with permission from Heidelberg Engineering GmbH).

Optical Coherence Tomography (OCT) is a high-resolution, non-invasive imaging method that can provide in-depth tomograms of biological tissue morphology in vivo. It is commonly used to precisely analyze retinal architecture and thickness, i.e., to diagnose and monitor retinal diseases [13]. OCT provides insight into the cross-sectional view of the retina and optic disc due to interference patterns produced by near-infrared light reflected from retinal tissue [14]. Near-infrared spectral range light waves, which penetrate several hundred microns into the tissue, are commonly used [13]. One of the possibilities provided by OCT is the creation of thickness maps. Predefined patterns like the *Early Treatment Diabetic Retinopathy Study* (ETDRS) grid patterns are commonly used to analyze obtained images (Figure 1). The ETDRS grid reduces the amount of data by exploiting only a few spatially averaged thickness grids from the presented thickness map in relation to nine sectors of the grid, i.e., the central foveal ring with a diameter of 1 mm, the inner macular ring (pericentral) with a diameter of 3 mm, and the outer macular ring (peripheral) with a diameter of 6 mm (Figure 1). The inner and outer rings are further divided into four quadrants (nasal, temporal, upper, and lower). Grid-based data analysis is commonly used to compare OCT findings [15]. An additional presentation of OCT analysis is the posterior pole asymmetry analysis (PPAA), which was primarily introduced for the purpose of detecting early glaucomatous damage (Figure 2). It consists of a square grid of 64 square areas covering the central 24° × 24° retina centered on the foveola. When examining the PPAA view, an insight into the thickness of each of the 64 square areas is obtained, as well as an insight into the retinal thickness asymmetry between the upper and lower corresponding square areas [16].

OCT devices have an axial resolution of a few microns and allow the accurate measurement of macular thickness [11,12]. An increase in the thickness and volume of the macula after PE, compared to preoperative values, is interpreted as a consequence of increased production of prostaglandins and other inflammatory mediators during PE. The accumulation in the aqueous humor and vitreous gel, and a direct effect on the permeability of the blood–retinal barrier, ultimately leads to the accumulation of fluid in the extracellular spaces of the macula lutea.

## 2. Materials and Methods

This is a prospective study [17,18] conducted in 2023 and 2024. The research was approved by the Ethics Committee of the Faculty of Medicine, J. J. Strossmayer University of Osijek (Class: 602-04/23-08/03; Reg. No: 2158-61-46-23-139).

Participants were patients planned for PE cataract surgery using the US according to inclusion and exclusion criteria at the “Ophthalmology Polyclinic Dr. Balog” in Osijek. The number of subjects was 79, i.e., the number of eyes on which cataract surgery was performed was 102.

Only otherwise healthy patients (without systemic diseases that can affect the eyes) with age-related cataracts and uneventful PE without any other eye diseases were included. Patients who used medications with a possible systemic impact on the eye (e.g., non-steroidal, anti-rheumatic drugs, corticosteroids, chloroquine, etc.) before surgery and during the follow-up period were excluded.

All included respondents signed the informed consent and consent for its implementation and data collection.

**Figure 3:**
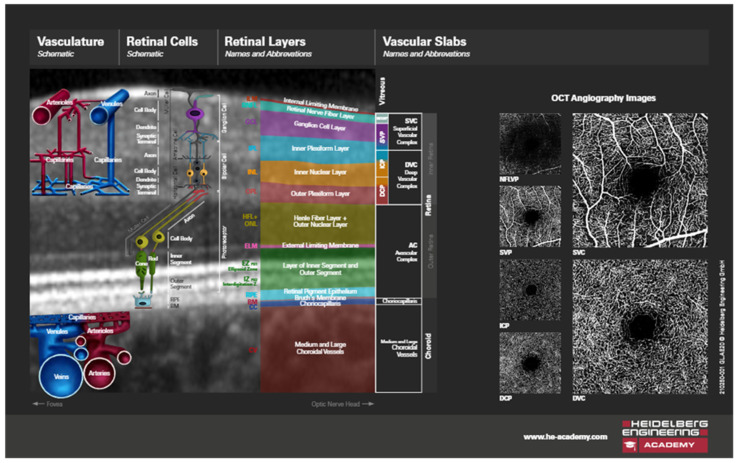
OCT image of a section through the retinal layers, schematic illustrations (published with permission from Heidelberg Engineering GmbH and courtesy of Mr. Chris Mody—Clinical Director, Posterior Eye Segment).

All participants underwent an ophthalmological examination, assessment of visual acuity, intraocular pressure, and biomicroscopic examination of the anterior and posterior segments of the eye. The thickness of the intraretinal layers measurement data expressed in microns was collected using the Spectralis HRA+OCT device (Heidelberg Engineering GmbH, Heidelberg, Germany). In this process, multiple-slice scans of the posterior pole, including the macular regions of the central retina, were taken (Figure 3). This device is equipped with an active eye-tracking system (i.e., TruTrack) to ensure precise positioning of OCT images. The OCT images of the patients were based on the PPAA method (Figure 2). Patients who were included in the regular program of cataract surgery at the Ophthalmology Polyclinic Dr. Balog in Osijek were operated on by the same surgeon. OCT images were taken before surgery and one week, one month, and three months after PE cataract surgery. A predetermined rectangular area scan pattern (volume scan) was 30° × 25° (8.3 × 6.9 mm) centered on the fovea. Such a predetermined imaging pattern is used to generate 61 consecutive PPAA B-scans at 115 µm intervals per participant. All OCT images were acquired using the high-speed imaging modality, with an axial and lateral resolution of 3.87 × 10.77 µm and a signal quality of at least 24 dB. Preoperative image quality was on average lower, i.e., it was at least 24 dB, while postoperatively it increased and reached up to 34 dB [19].

**Figure 4:**
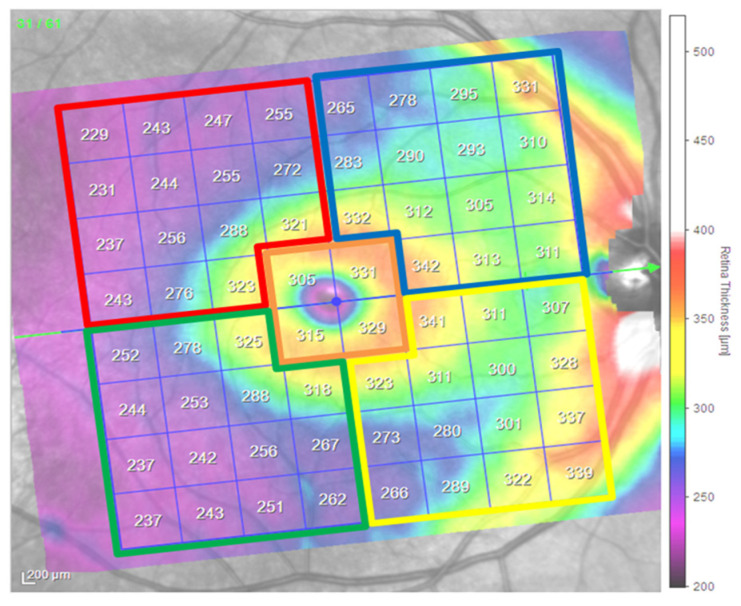
PPAA view and division by selected segments of the right eye, schematic illustrations (published with permission from Heidelberg Engineering GmbH). Legend of PPAA areas and their color coding: inferior temporal area of the macula (IT)—green, inferior nasal area of the macula (IN)—yellow, superior temporal area of the macula (ST)—red, superior nasal area of the macula (SN)—blue, central macular area (CMA)—orange.

The volume datasets of all participants were further processed using device-specific software (Heidelberg Eye Explorer, HEYEX version 6.13.3.0) to perform retinal layer segmentation. Finally, average thickness values were obtained per participant and per retinal layer, reflecting the entire posterior pole, including the macular as well as the foveal region of the PPAA (Figure 4, Table 1).

PE surgery was performed using a 2.4 mm clear corneal incision (CCI) performed in the upper quadrants of the cornea, in the 11 to 13 o’clock position.

During this research, an intraocular lens from a renowned manufacturer was used (this included the following characteristics: posterior chamber hydrophobic acrylic single-piece intraocular lens, 6 mm in diameter, biconvex, square-edged optic, 13 mm in overall diameter, with a zero-degree angle of haptic–optic configuration, a water content <5%, and a refractive index of 1.5; the material is acrylic and hydrophobic), and the IOL was implanted in the capsular bag.

The standard PE technique consists of using a 2.4 mm CCI without combining it with femtosecond or any other surgical method.

The values of US energy used during cataract surgery were read from the Oertli Faros cataract surgery device after the surgical procedure was performed on each patient. It is important to note that the same anti-inflammatory drugs and regimen were used in all patients during the study.

In the technical data for all Oertli devices (manufacturer), namely, the Oertli easyPhaco handpiece, one can find the common feature of six piezoelectric crystals, and they are all unique in terms of HF output power Phaco, in the range of 26–30 KHz, with a normative value of 28 KHz [20].

The PSPP v.3 software was used for data analysis. Correlation, *t*-test for independent samples, and analysis of variance for dependent samples were used. The defined level of significance is *p* < 0.05. The normality of the distributions was checked using the Kolmogorov–Smirnov test. Most of the variables showed a normal distribution, while the others were normalized in order to use parametric statistics. Descriptive data are presented tabularly and graphically, and the arithmetic mean and standard deviation are used to present data. The independent variable in the study was the amount of applied US energy, while the dependent variables were changes in five areas of the macula through 10 retinal layers. A significance level of 5% was used in this research. The minimum required number of respondents was 67 (defined by program G*Power 3.1) with an effect size of 0.3 and a test power of 0.80 [21].

## 3. Results

### 3.1. Basic Characteristics of the Participants

In total, 102 eyes (57 (58.8%) right eyes and 45 (44.2%) left eyes) of 79 patients were examined in the study. There were 46 (58%) women and 33 (42%) men in the sample. The average age of participants was 68.87 ± 11.20 years of age. The average age was 65.36 ± 10.94 in male participants and 71.39 ± 11.20 in female participants.

### 3.2. The Thickness of the Selected Layers of the Retina

**Figure 5:**
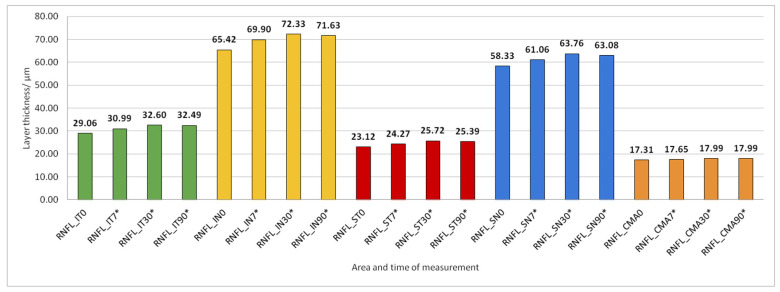
Retinal nerve fiber layer (RNFL) thickness preoperatively (0) and 7, 30, and 90 days after phacoemulsification. Statistically significant differences in the change of layer thickness compared to initial values are marked by an asterisk (*).

**Figure 6:**
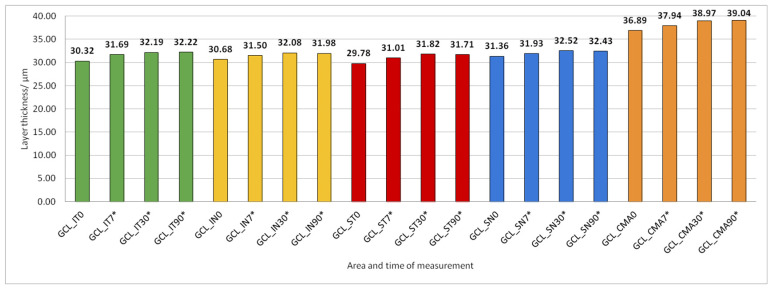
Ganglion cell layer (GCL) thickness preoperatively (0) and 7, 30, and 90 days after phacoemulsification. Statistically significant differences in the change of layer thickness compared to initial values are marked by an asterisk (*).

**Figure 7:**
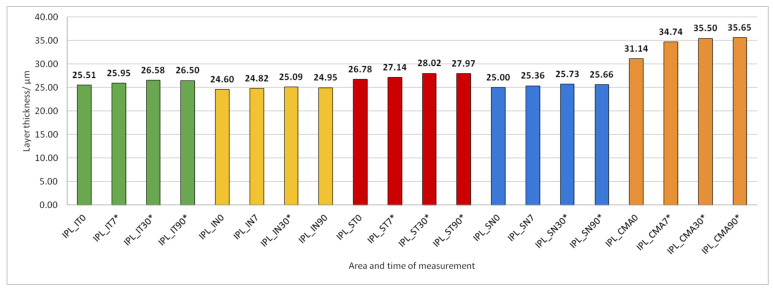
Inner plexiform layer (IPL) thickness preoperatively (0) and 7, 30, and 90 days after phacoemulsification. Statistically significant differences in the change of layer thickness compared to initial values are marked by an asterisk (*).

**Figure 8:**
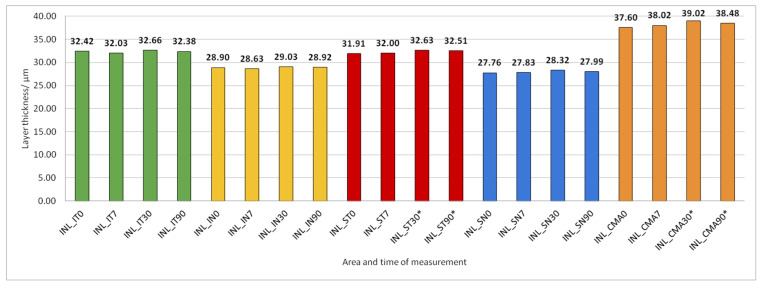
Inner nuclear layer (INL) thickness preoperatively (0) and 7, 30, and 90 days after phacoemulsification. Statistically significant differences in the change of layer thickness compared to initial values are marked by an asterisk (*).

**Figure 9:**
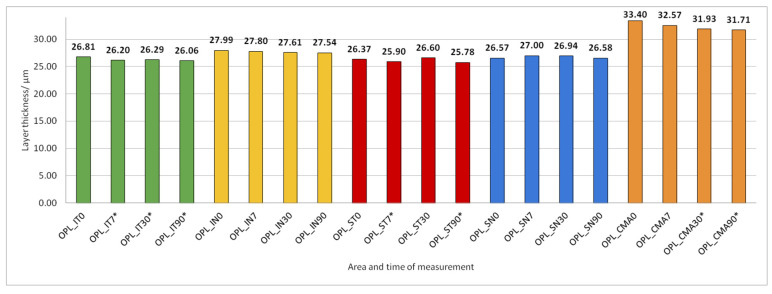
Outer plexiform layer (OPL) thickness preoperatively (0) and 7, 30, and 90 days after phacoemulsification. Statistically significant differences in the change of layer thickness compared to initial values are marked by an asterisk (*).

**Figure 10:**
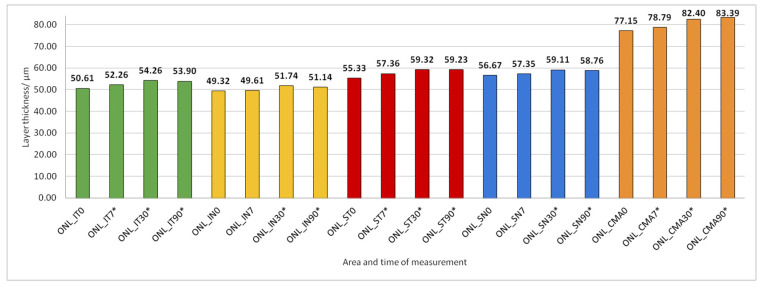
Outer nuclear layer (ONL) thickness preoperatively (0) and 7, 30, and 90 days after phacoemulsification. Statistically significant differences in the change of layer thickness compared to initial values are marked by an asterisk (*).

**Figure 11:**
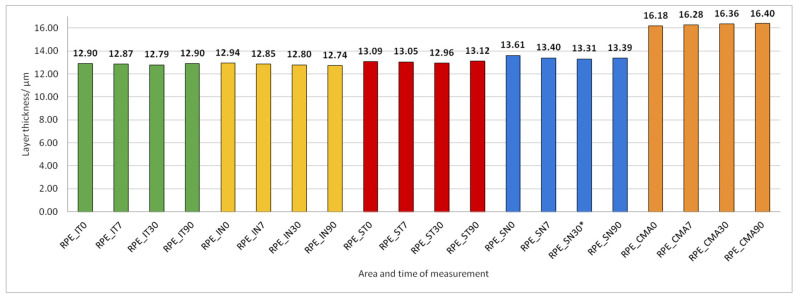
Retinal Pigment Epithelium (RPE) layer thickness preoperatively (0) and 7, 30, and 90 days after phacoemulsification.

**Figure 12:**
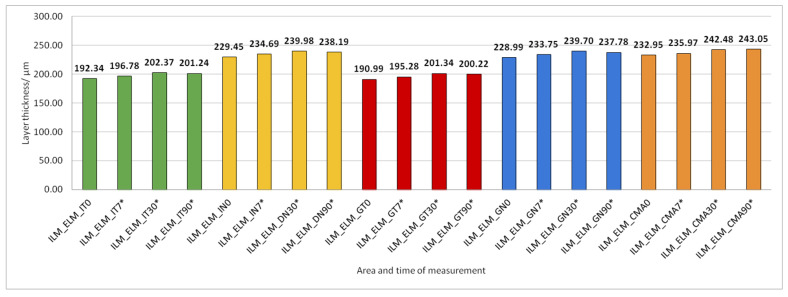
The thickness of the inner retinal layers from the internal limiting membrane (ILM) layer to the external limiting membrane (ELM) layer preoperatively (0) and 7, 30, and 90 days after phacoemulsification. Statistically significant differences in the change of layer thickness compared to initial values are marked by an asterisk (*).

**Figure 13:**
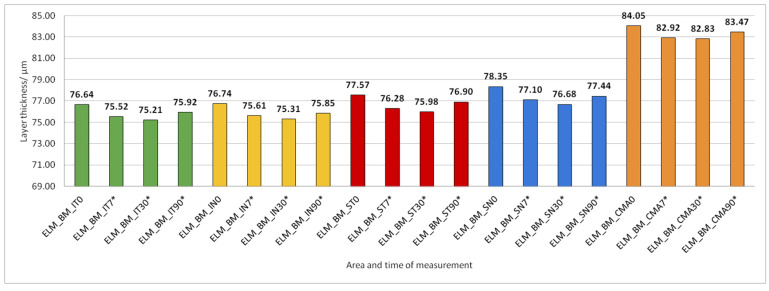
The thickness of the external retinal layers from the ELM layer to Bruch’s membrane (BM) preoperatively (0) and 7, 30, and 90 days after phacoemulsification. Statistically significant differences in the change of layer thickness compared to initial values are marked by an asterisk (*).

**Figure 14:**
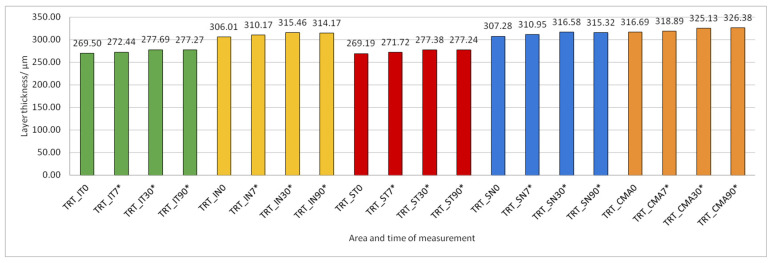
Total retinal thickness (TRT) preoperatively (0) and 7, 30, and 90 days after phacoemulsification. Statistically significant differences in the change of layer thickness compared to initial values are marked by an asterisk (*).

Data on how a particular intraretinal layer in a predetermined part of the macula changed during the time after PE are presented in Figure 5, Figure 6, Figure 7, Figure 8, Figure 9, Figure 10, Figure 11, Figure 12, Figure 13 and Figure 14. Within Figure 5, Figure 6, Figure 7, Figure 8, Figure 9, Figure 10, Figure 11, Figure 12, Figure 13 and Figure 14, statistically significant differences in the change of layer thickness compared to initial values are marked by an asterisk (*).

The thickness of the inner retinal layers from the internal limiting membrane layer to the external limiting membrane (ILM-ELM), thickness of the outer retinal layers from the ELM layer to Bruch’s membrane (ELM-BM), as well as total retinal thickness (TRT), RNFL and GCL thickness increased significantly in all predetermined parts of the macula at all time points after PE (Figure 5, Figure 6, Figure 12, Figure 13 and Figure 14).

IPL thickness increased significantly in all predetermined parts of the macula but not at all time points after PE (Figure 7).

INL thickness increased significantly in SN and CMA 30 and 90 days after PE (Figure 8).

After PE, OPL thickness increased significantly in IT, ST, and CMA (Figure 9).

ONL thickness increased significantly in all predetermined parts of the macula 30 and 90 days after PE (Figure 10).

RPE thickness did not change during the 90 days after PE (Figure 11).

There is a statistically significant correlation between the US energy used and the increase in thickness of certain layers and areas of the macula. All statistically significant correlations of the used US during PE and thickness of the observed layers and macular area are small and negative. A significant correlation of used US energy during PE was observed with the change in RNFL thickness from days 0 and 90 after surgery in CMA and GCL in the SN area; IPL in ST; SN and CMA; ELM in CMA; and total retinal thickness (TRT) in the ST and SN area compared to initial layer thickness. There is no statistical connection with the other layers and areas.

Based on the results in Table 2, we can see the existence of statistical significance in the relationship between the spent US energy and the obtained total difference in the thickness of certain layers and areas of the macula. In Table 2, the existence of moderate, positive correlations is noticeable. Looking at the areas and layers, negative correlations were observed in the layers and areas of RNFL CMA, INL SN and INL IN, OPL CMA, ONL SN, ONL ST, ELM-BM SN, and ELM-BM ST, which shows that there is a connection between the increase in the amount of US energy and the decrease in the thickness layer. On the other hand, a positive correlation is visible in the layer and area of GCL SN and GCL IT, which shows us that, in these two areas, there is a connection between the increase in the amount of US energy used and the increase in the thickness of the layer.

## 4. Discussion

We recorded US energy used during uneventful PE cataract surgery, measured postoperative changes in the thickness of individual intraretinal layers in five defined areas of the macula, and correlated these values. We compared our results with the results of similar results obtained by other researchers.

Some previous studies have also documented changes in the thickness of individual retinal layers, but only a few in different areas of the macula. In our study, we have found a significant increase in the total macular thickness (ILM-ELM), but predominantly of the inner retinal layers, primarily at the level of RNFL, GCL, and IPL, but also of the ONL layer in all macular sectors after PE. We presume that inflammatory factors induced by PE may play a role in that process [22]. Similarly to our study, in a study conducted by Schwarzenbacher et al., which monitored changes in the retinal layers 18 months after femtosecond laser-assisted cataract surgery and manual cataract surgery, a statistically significant increase in the thickness of the INL, ONL, and TRT was observed; however, there was no increase in the thickness of the OPL layer. Since the subjects were followed for 18 months, it was observed that there was a discrete decrease in the thickness of the outer retinal (non-vitreous) layers after 6 weeks and 18 months following the surgery; however, the macular thicknesses still did not return to baseline values. Overall, the macula remained statistically significantly thicker compared to baseline values in the perifoveal zone from 1 to 3 mm [23]. We did not find a significant increase in the thickness of the INL and OPL layers in all macular sectors, but only in the CMA and ST areas of the macula 90 days after PE. This phenomenon can be explained by the position and manner in which the surgeon holds the phacoemulsification probe during PE surgery [24]. The increase in the INL layer thickness was also noted in some other clinical entities, like optic neuritis related to multiple sclerosis, but was associated with the severe loss of GCL layer cells [25].

Based on previous research, it is evident that the RPE layer thins as an individual ages [26]. We did not find any significant changes in RPE thickness during the period of 90 days after PE.

The only retinal layer that showed a significant decrease in thickness in all macular areas 7 to 90 days after PE was the ELM-BM layer, and we presume that it might be a consequence of photoreceptor loss due to surgery. Furthermore, we found a negative correlation of US energy used during PE with ELM-BM layer thickness change, which was the most significant in superior macular areas, which are closer to the US probe during PE surgery. Therefore, we believe that higher US energy used during the PE negatively affects macular photoreceptors. Another analysis, one conducted by Kamal Abdellatif et al., also noticed that the superior and inferior parafoveal and perifoveal areas were probably affected by age, but not by choroidal thinning. The loss of neural tissue associated with photoreceptor aging probably has a more pronounced impact on outer retinal thickness than the reduction in vascular supply due to choroidal thinning. Therefore, we would like to emphasize the importance of considering the patient’s age in predicting visual prognosis and clinical state after PE [26]. Depending on the thinning of the RPE, this may also be a prognostic factor for the development of macular disease [27].

The comparison of TRT values after PE obtained in this research and the TRT values in similar articles was made. Also, given that the studied areas of the macula in our research are defined as in Figure 2, the selected CMA area on the PPAA image covers a wider area than the inner circle of the ETDRS image that was used in previous studies to determine CMA thickness. As expected, the average values obtained for CMA on the PPAA view are, therefore, higher than the standard; however, the increase in retinal thickness after PE and correlations of retinal thickness with US time during PE were in accordance with the previous studies.

Most correlations of US time spent during PE with the change in thickness in distinct retinal layers 90 days after PE in our research were not statistically significant. Previous research has been conducted on several different devices. Thus, Bambo, Garcia-Marin, Otin et al. decided to compare the values of macular and RNFL measurements in healthy subjects one month before surgery and one month after PE surgery on two devices (Cirrus OCT and Spectralis OCT [14]), as did Rodriguez-Mena, Dolza et al., who additionally observed the thickness of the RNFL layer [13]. When conducting their research, the aforementioned authors did not observe changes in the macular thickness measured using the Spectralis OCT device that we also used in our research; however, when using the Cirrus OCT, they came to different conclusions. In the research conducted by Bambo, Garcia-Martin, Otin et al., it was observed that all parameters in the macula become statistically significantly thickened one month after the surgery. The biggest difference was observed in the change in the thickness of the fovea, which was 60 µm thicker after the PE (*p* = 0.006) [14]. We also realized that all measured areas of the macula significantly thickened, and the most significant difference was found within the inner layers of the retina from the ILM to the ELM layer.

Celik, Cakir, Turkoglu et al. observed a statistically significant increase in the thickness of the layer of ganglion cells and the RNFL layer after PE surgery. The stated values support our results.

According to our measurements, there is also an increase in the thickening of the central retinal area according to the PPAA view. In relation to the preoperatively measured values of 316.69 ± 16.312 µm, after one week, thickness values of 318.88 ± 16.487 µm were measured; after one month, they measured 325.13 ± 18.553 µm, while after three months, the increased value remained, at 326.37 ± 19.232 µm. Also, in the research conducted by Anastasilakis, Mourgel, Symeonidis et al., an increase in the central foveal thickness was monitored after one month and after three months, compared to the measured preoperative values. The preoperative thickness of the retina in the area of the fovea in their research was 251.51 ± 20.2 µm, while one month postoperatively it was 257.00 ± 22.3 µm, and three months after the operation it was 255.92 ± 21.6 µm. In our study, three months after surgery, the value of retinal thickness was still higher than one month postoperatively, and in the aforementioned study, these values decreased after three months. According to the available literature, cystoid macular edema (CME) most often occurs postoperatively due to certain trauma caused by the surgical procedure, i.e., if an attempt were made to reduce the frequency of intraoperative complications and reduce the amount of PE energy used, the incidence of CME would consequently be lower. Given that inflammatory factors are thought to contribute to increased permeability of the macular blood–retinal barrier, which ultimately leads to CME, with an accumulation of fluid in the extracellular spaces, the use of techniques in surgery that reduce the amount of released US energy during PE could result in reduced risk for the development of CME [28].

The technique of the PE is individual and specific to the operator, who has his own way of holding the PE probe, directing the PE probe, and performing the PE, as well as the method and technique of controlling the probe and delivering the US energy. Including more surgeons in future studies and taking into consideration the position of the PE probe in the surgeon’s hand might give us insight into whether the US energy used during PE has any effects on the localization of changes in the macular area.

It would be desirable to repeat this type of research, but also with the use of OCT angiography, to gain insight into how much vascular components (vascular density parameters as well as FAZ) influence or can potentially contribute to this type of changes, both in the center of the macula and on its periphery, especially because the choroid is known to have a thermoregulatory effect on the overlying retina.

Additional restrictions we have identified are the poor quality of the OCT image in subjects with a thick cataract and the poor quality of the tear film. Consequently, the lines that determine the thickness on the OCT image are not always automatically drawn correctly, but sometimes need to be manually corrected. Also, different positions of clear corneal incisions and habits of holding the PE probe depend on the surgeon during surgery (and it might affect the results of the study). In addition to that, uncertainty in reading values from the PE device, the varied duration of the operation, as well as the amount of fluid flow during PE are cited as additional limitations [4,29,30]. Of course, with the extended duration of the operation and use of higher ultrasound energy, a more pronounced inflammatory response of the eye can be expected, and it is also essential to mention the role of the use of non-steroidal anti-inflammatory drugs in preoperative and postoperative therapy. All the mentioned factors influence to some extent the final results of this research, which should certainly be analyzed in more detail in subsequent research, on a larger sample.

## 5. Conclusions

We found a significant increase in total retinal thickness in the macula as well as an increase in certain retina layer thickness 7 to 90 days after uneventful PE, which was most evident in inner retinal layers (RNFL, GCL, and IPL in all macular areas and INL in CMA and ST area), but also in outer retinal layers (ONL in all macular areas and OPL in CMA, IT, and ST areas). Postoperative inflammation may play a role in that process.

The only retinal layer that showed a significant thickness decrease in all macular areas 7 to 90 days after PE was the ELM-BM layer, and we presume that it might be a consequence of photoreceptor loss due to surgery.

Nevertheless, the results could also be a product of a measurement error because the measurement was first made through a crystalline lens and then through a plastic intraocular lens.

A negative correlation of the US energy used during PE with INL, ONL, and ELM-BM layer thinning 90 days after PE suggests that higher US energy used during PE negatively affects macular photoreceptors and bipolar cells. On the contrary, a positive correlation of US energy used during PE with GCL 90 days after PE may be a result of inflammation. Total retinal thickness change after PE did not correlate with the US energy used during surgery and is, therefore, not a good method of measuring the effect of US on the macula after PE.

We have shown that there are some interconnections between the US energy spent during PE and the change in the thickness of macular layers.

Further, a longer analysis of that effect should be performed, but a layered analysis like in our study is suggested.

## Figures and Tables

**Table 1:** Legend of PPAA areas and their color coding depending on the measurement period in days: inferior temporal (IT) area of the macula—green, inferior nasal (IN) area of the macula—yellow, superior temporal (ST) area of the macula—red, superior nasal (SN) area of the macula—blue, central macular area (CMA)—orange.

Region/Days	IT	IN	ST	SN	CMA
0 (preoperatively)	IT0	IN0	ST0	SN0	CMA0
7 (postoperatively)	IT7	IN7	ST7	SN7	CMA7
30 (postoperatively)	IT30	IN30	ST30	SN30	CMA30
90 (postoperatively)	IT90	IN90	ST90	SN90	CMA90

**Table 2:** Pearson’s correlation coefficient of the US energy used during PE with the difference in the total retinal thickness between the first and last measurements (90th day).

	IT	IN	ST	SN	CMA
ILM-ELM	−0.068	−0.079	−0.089	−0.097	−0.063
RNFL	0.028	0.086	0.042	−0.121	−0.349 **
GCL	0.207 *	0.162	0.176	0.214 *	−0.011
IPL	0.069	−0.108	0.08	−0.023	−0.021
INL	−0.163	−0.248 *	−0.052	−0.204 *	0.056
OPL	0.014	−0.184	0.039	−0.083	−0.254 **
ONL	−0.149	−0.077	−0.249 *	−0.219 *	0.106
ELM-BM	−0.163	−0.184	−0.305 **	−0.210 *	−0.065
RPE	0.135	0.139	0.112	0.144	0.125
TRT	−0.094	−0.08	−0.149	−0.121	−0.061

* *p* < 0.05, ** *p* < 0.01.

## Data Availability

All data are available and can be delivered to anyone upon request.

## References

[1] Cicinelli M.V., Buchan J.C., Nicholson M., Varadaraj V., Khanna R.C. (2023). Cataracts. Lancet.

[2] Olson R.J. (2018). Cataract Surgery From 1918 to the Present and Future-Just Imagine!. Am. J. Ophthalmol..

[3] Gharbiya M., Cruciani F., Cuozzo G., Parisi F., Russo P., Abdolrahimzadeh S. (2013). Macular thickness changes evaluated with spectral domain optical coherence tomography after uncomplicated phacoemulsification. Eye.

[4] Pardianto G., Moeloek N., Reveny J., Wage S., Satari I., Sembiring R., Srisamran N. (2013). Retinal thickness changes after phacoemulsification. Clin. Ophthalmol..

[5] Pašová P., Skorkovská K. (2016). The Effect of Cataract Surgery on the Reproducibility and Outcome of Optical Coherence Tomography Measurements of Macular and Retinal nerve Fibre Layer Thickness. Cesk. Slov. Oftalmol..

[6] Liu J., Liu Q., Yu H., Xia Y., Zhang H., Geng C., Dong L. (2021). Microvascular Changes in Macular Area After Phacoemulsification and Its Influencing Factors Assessed by Optical Coherence Tomography Angiography. Ther. Clin. Risk Manag..

[7] Zhao Z., Wen W., Jiang C., Lu Y. (2018). Changes in macular vasculature after uncomplicated phacoemulsification surgery: Optical coherence tomography angiography study. J. Cataract. Refract. Surg..

[8] Lobo C.L., Faria P.M., Soares M.A., Bernardes R.C., Cunha-Vaz J.G. (2004). Macular alterations after small-incision cataract surgery. J. Cataract. Refract. Surg..

[9] Pacifico R.L. (1994). Ultrasonic energy in phacoemulsification: Mechanical cutting and cavitation. J. Cataract. Refract. Surg..

[10] Abd El-Mawgoud S., Arfeen Shaimaa A.S., El-Gendy N., Fathy A. (2018). Early changes of choroidal and macular thickness after uneventful phacoemulsification surgery. Delta J. Ophthalmol..

[11] Zhou Y., Zhou M., Wang Y., Ben S., Gao M., Zhang S., Liu H., Sun X. (2020). Short-Term Changes in Retinal Vasculature and Layer Thickness after Phacoemulsification Surgery. Curr. Eye Res..

[12] Kim B.J., Ahn Y.J., Oh H.Y., Choi S.I., Yoo Y.S., Whang W.J., Byun Y.-S., Lee M.-Y., Joo C.-K. (2022). Assessment for Macular Thickness after Uncomplicated Phacoemulsification Using Optical Coherence Tomography. Korean J. Ophthalmol..

[13] Garcia-Martin E., Rodriguez-Mena D., Dolz I., Almarcegui C., Gil-Arribas L., Bambo M.P., Larrosa J.M., Polo V., Pablo L.E. (2013). Influence of cataract surgery on optical coherence tomography and neurophysiology measurements in patients with retinitis pigmentosa. Am. J. Ophthalmol..

[14] Bambo M.P., Garcia-Martin E., Otin S., Sancho E., Fuertes I., Herrero R., Satue M., Pablo L. (2014). Influence of cataract surgery on repeatability and measurements of spectral domain optical coherence tomography. Br. J. Ophthalmol..

[15] Prakasam R.K., Röhlig M., Fischer D.C., Götze A., Jünemann A., Schumann H., Stachs O. (2019). Deviation Maps for Understanding Thickness Changes of Inner Retinal Layers in Children with Type 1 Diabetes Mellitus. Curr. Eye Res..

[16] Dave S.D., Zeppieri M., Meyer J.J. (2025). Chronic Closed Angle Glaucoma. StatPearls [Internet].

[17] Kločić I., Vorko-Jović A. (2012). Epidemiologija.

[18] Marušić M. (2013). Uvod u Znanstveni rad u Medicine.

[19] Wojtkowski M., Srinivasan V., Fujimoto J.G., Ko T., Schuman J.S., Kowalczyk A., Duker J.S. (2005). Three-dimensional retinal imaging with high-speed ultrahigh-resolution optical coherence tomography. Ophthalmology.

[20] (2012). Operating Manual & ParaProg Manual FarosTM, VC840100/VC840101, VV016034E.

[21] Ivanković Det A.L. (1988). Osnove Statističke Analize za Medicinare.

[22] Predović J., Bosnar D., Marković L., Ćurić A., Bračić J., Georgi T., List W., Glatz W., Ivastinovic D. (2024). Vitreous hyper-reflective dots and the macular thickness after cataract surgery. PLoS ONE.

[23] Schwarzenbacher L., Schmidt-Erfurth U., Schartmüller D., Röggla V., Leydolt C., Menapace R., Reiter G.S. (2024). Long-term impact of low-energy femtosecond laser and manual cataract surgery on macular layer thickness: A prospective randomized study. Acta Ophthalmol..

[24] Jha B., Sharma R., Vanathi M., Agarwal T., Sidhu T., Tomar A., Dada T. (2017). Effect of phacoemulsification on measurement of retinal nerve fiber layer and optic nerve head parameters using spectral-domain-optical coherence tomography. Oman J. Ophthalmol..

[25] Kaushik M., Wang C.Y., Barnett M.H., Garrick R., Parratt J., Graham S.L., Sriram P., Yiannikas C., Klistorner A. (2013). Inner nuclear layer thickening is inversely proportional to retinal ganglion cell loss in optic neuritis. PLoS ONE.

[26] Kamal Abdellatif M., Abdelmaguid Mohamed Elzankalony Y., Abdelmonsef Abdelhamid Ebeid A., Mohamed Ebeid W. (2019). Outer Retinal Layers’ Thickness Changes in relation to Age and Choroidal Thickness in Normal Eyes. J. Ophthalmol..

[27] Zekavat S.M., Sekimitsu S., Ye Y., Raghu V., Zhao H., Elze T., Segrè A.V., Wiggs J.L., Natarajan P., Del Priore L. (2022). Photoreceptor Layer Thinning Is an Early Biomarker for Age-Related Macular Degeneration: Epidemiologic and Genetic Evidence from UK Biobank OCT Data. Ophthalmology.

[28] Anastasilakis K., Mourgela A., Symeonidis C., Dimitrakos S.A., Ekonomidis P., Tsinopoulos I. (2015). Macular edema after uncomplicated cataract surgery: A role for phacoemulsification energy and vitreoretinal interface status?. Eur. J. Ophthalmol..

[29] Benjamin L. (2018). Fluidics and rheology in phaco surgery: What matters and what is the hype?. Eye.

[30] Gudauskienė G., Povilaitytė I., Šepetauskienė E., Žaliūnienė D. (2020). Phacoemulsification Induced Changes of Choroidal Thickness in Eyes with Age-Related Macular Degeneration. Medicina.

